# Supra-Pubic Cystotomy, with Removal of Oxalate of Lime Stone

**Published:** 1892-10

**Authors:** Jas. S. White

**Affiliations:** Columbia, Ky.


### SUPRA-PUBIC CYSTOTOMY, WITH REMOVAL OF OXALATE OF LIME STONE.

By JAS. S. WHITE, M. D.,
COLUMBIA, KY.

Patient, girl, aged eight years and six months. After careful examination of the case in question, and knowing an operation imperative, I deemed it expedient to call a consultant. Accordingly my friend, Dr. Jno. Grady, visited the patient, whom we found suffering excruciatingly from those peculiar lancinating pains so common in urinary calculus, except when dulled by opiates. Owing to the patient's fear of anæsthetics, we attempted introduction of sound without it.

Frequent micturition of exceedingly acid urine had excoriated the parts to such a degree that even touching the external genitals was attended with excruciating pain. A few minutes being spent in this fruitless endeavor, chloroform narcosis was decided on and induced in a short time. A metal instrument having been passed into the bladder, that distinctive "feel" so characteristic of present urinary calculi was at once pronounced. Locating the offending body we made known the facts to the parents; partial steps to the operation were explained, and their consent gained. Considering lithotrity the proper safe means then at our command a day for the operation was chosen. Delay in getting the necessary instruments, and gradual weakening condition of the patient, called for immediate action. Accordingly, after thoroughly clean preliminaries, sleep was produced and the operation made. A curved sound was introduced into the bladder, and the point strongly forced into "view" immediately above the os pubis. An incision one and three quarter inches in length was made in the median line, as nearly as possible to the pubic bone, through the several tissues to the bladder wall. Retracting these to expose more surface of the underlying viscus, a tenaculum holding this in position, suture silk was passed on opposite sides of the tenaculum for the two-fold purpose of supporting and, after final incision, retracting edges of wall. A small opening having been made through the bladder wall I introduced my left index finger, thereby further dilating the cut, and experiencing the "button-hole" sensation of Wm. Hunt, M. D. The stone was to a degree encysted in the *bas fond* of bladder. Loosening it with my finger, and being guided in the same way, I passed forceps into cavity and removed stone. With its removal the anæsthetic was stopped, and the abdominal and bladder openings were flushed with hot boracic acid solution till the latter came away perfectly clear. Then suturing edges of bladder wall to the corresponding edges of abdominal wall (two sutures to each side of opening), we completed dressing with borated absorbent cotton saturated with fresh boracic acid, over that a compress, and around the body the indispensable "binder." Then prescribed

℞. Quinia sul., . . . gr. ii

Morphia sul., . . . gr. ⅛

to be given every three hours. The patient having in the meantime recovered consciousness was left to the care of the family, to be seen by us a few hours later.

On July 25th, twelve hours after operation, pulse rate 128 per minute; temperature 100 2–5; July 26th, morning pulse 120; temperature 99 2–5; July 26th, evening pulse 120; temperature 100. From this date improvement was so marked and rapid as to be noted even by the parents; hence, continuing the same course of treatment until afternoon of fourth day, I found union so far advanced as to be enabled to remove two sutures, and on the second day following the remaining two were taken; when the floor of the incision or bladder wall was seen to be healed an application of iodoform was then made.

By means of this agent the granulation was so rapid that on August 3d, ten days after the operation, recovery was regarded as complete; and indeed not one untoward symptom presented afterward. From second day after operation urine flowed perfectly clear and normal save slight acidity, which was easily corrected by use of acetate of potash in ten grain doses three times a day.

